# Ethiopia’s transforming wheat landscape: tracking variety use through DNA fingerprinting

**DOI:** 10.1038/s41598-020-75181-8

**Published:** 2020-10-28

**Authors:** D. P. Hodson, M. Jaleta, K. Tesfaye, C. Yirga, H. Beyene, A. Kilian, J. Carling, T. Disasa, S. K. Alemu, T. Daba, A. Misganaw, K. Negisho, Y. Alemayehu, A. Badebo, B. Abeyo, O. Erenstein

**Affiliations:** 1 International Maize and Wheat Improvement Center (CIMMYT), Mexico City, Mexico; 2 International Maize and Wheat Improvement Center (CIMMYT), Addis Ababa, Ethiopia; 3 Ethiopian Institute of Agricultural Research (EIAR), Addis Ababa, Ethiopia; 4 Ethiopian Institute of Agricultural Research (EIAR), Holeta, Ethiopia; 5 Ethiopian Central Statistical Agency (CSA), Addis Ababa, Ethiopia; 6 Diversity Array Technologies (DArT), Canberra, Australia

**Keywords:** Agricultural genetics, Genotype, Plant breeding

## Abstract

Ethiopia is the largest wheat producer in sub-Saharan Africa yet remains a net importer. Increasing domestic wheat production is a national priority. Improved varieties provide an important pathway to enhancing productivity and stability of production. Reliably tracking varietal use and dynamics is a challenge, and the value of conventional recall surveys is increasingly questioned. We report the first nationally representative, large-scale wheat DNA fingerprinting study undertaken in Ethiopia. Plot level comparison of DNA fingerprinting with farmer recall from nearly 4000 plots in the 2016/17 season indicates that only 28% of farmers correctly named wheat varieties grown. The DNA study reveals that new, rust resistant bread wheat varieties are now widely adopted. Germplasm originating from CGIAR centres has made a significant contribution. Corresponding productivity gains and economic benefits have been substantial, indicating high returns to investments in wheat improvement. The study provides an accurate assessment of wheat varietal status and sets a benchmark for national policy-makers and donors. In recent decades, the Ethiopian wheat landscape has transformed from local tetraploid varieties to widespread adoption of high yielding, rust resistant bread wheat. We demonstrate that DNA fingerprinting can be applied at scale and is likely to transform future crop varietal adoption studies.

## Introduction

Wheat is an important staple food crop in Ethiopia and since 2005 the country has been the largest producer of wheat in sub-Saharan Africa^1^. Wheat is grown on 1.6–1.8 million ha annually, with an estimated 5 million farming household’s dependant on the crop^2^. Wheat serves as both a food crop and an important source of income for Ethiopian small-holders. Demand for wheat is growing rapidly in Ethiopia, reflecting population growth and shifting dietary patterns linked to urbanization that are mirrored across other eastern and southern African countries^3^. Ethiopia remains a net importer of wheat, meeting just over 70% of demand from domestic production^4^. However, there are signs that the wheat sector in Ethiopia is undergoing a significant transformation and productivity is increasing rapidly. Despite these recent productivity gains, shortfalls remain and drastically narrowing the gap between supply and demand to enhance (and possibly achieve) self-sufficiency in wheat production is a high national priority. Food security issues and the need to reduce spending of scarce foreign currency reserves on costly wheat imports are both of paramount importance to the Government of Ethiopia.

Farmers in Ethiopia grow both bread and durum wheat. Ethiopia is known as a center of diversity for tetraploid wheat including durum wheat^5,6^, although recent genetic analysis from Kabbaj et al.^7^ indicated that Ethiopia might actually represent a second center of origin for durum wheat. No official national statistics are maintained on the proportion of bread and durum wheat in Ethiopia, but expert opinion and survey data indicates a significant shift towards bread wheat in recent decades. Until the mid-1980s, durum wheat dominated the wheat production in Ethiopia. Tesemma^8^ estimated that 60–70% of the total wheat area was planted to durum wheat and traditional landraces dominated. Improved durum wheat varieties were estimated to only account for 10% of this area. With the introduction of high yielding semi-dwarf bread wheat varieties, the area planted to durum wheat in Ethiopia has declined significantly. In 2009/10, the Diffusion and Impact of Improved Varieties in Africa (DIIVA) project estimated the area planted to durum wheat in Ethiopia to have declined to 17% of which virtually all (99.5%) were considered to be local varieties^9^. No subsequent estimate of the extent of durum wheat in Ethiopia is currently available.

For decades wheat productivity in Ethiopia remained stagnant at very low levels (SI Figure 1). Reported national average wheat yields in 2000/01 were only 1.16 t/ha^1^, little higher than in 1975 (0.91 t/ha). Contributing factors to this low productivity included: the use of low yielding traditional wheat varieties^10,11^, sub-optimal tillage practices^12^, abiotic and biotic stresses (notably drought and wheat rust diseases), and limited use of inputs such as inorganic fertilizer and fungicides^13,14^. Lack of a developed seed system and limited access to credit are also important constraints. However, in the last 15 years there are signs of dramatic change. In 2016/17 national average wheat yields were reported to be 2.68 t/ha^1^, a twofold wheat productivity increase since the turn of the century (SI Figure 1).

There are both formal and informal wheat seed systems in Ethiopia, where the informal system is dominating up to 80% of the seed delivery to smallholder farmers^15^. The formal seed system is mostly used in popularizing newly released wheat varieties which later would be taken up by the informal seed system where farmer-to-farmer seed exchange, using own saved seeds, buying from local markets, etc. are the common wheat seed sources. New varieties are popularized through on-farm and on-station demonstrations and farmers’ field days. To increase the seed quantity of newly released varieties, pre-basic and basic seeds are distributed to public and private seed enterprises and cooperatives for a wider seed multiplication and marketing to smallholder farmers. The recent initiatives by different projects on fast-track pre-release seed multiplication contributed much in making breeders’ seed available in large volume for seed multiplication at the time of varietal release and enabled to overcome the usual 2–10 years delay between varietal release and seed deployment to farmers^16,17^. Demonstrations at Farmers’ Training Centres by the Agricultural extension system under the Ministry of Agriculture and the recent cluster approach adopted by the Ethiopian Institute of Agricultural Research (EIAR) and the Agricultural Transformation Agency (ATA) in demonstrating newly released improved varieties at large scale played key roles in popularizing newly released improved wheat varieties. Moreover, farmers neighbouring to wheat research centres also get quality seeds of newly released varieties to use and disseminate to their neighbours and fellow farmers.

Wheat rust (fungal) diseases, notably stem (black) and stripe (yellow) rust, are the most important biotic constraints to wheat production in Ethiopia^18,19^. Recurrent rust epidemics have caused large scale production losses in recent years^20,21^. The emergence and incursion of new, virulent wheat rust races poses a significant and continuing threat to Ethiopian wheat production. As a result, wheat improvement programs in Ethiopia have prioritized rust resistance as a key trait in the development of new, improved varieties. The breeding and dissemination of new improved varieties in Ethiopia is seen as an essential component of agricultural development. The Ethiopian national and regional wheat programs, in partnership with international agricultural research centres such as the International Maize and Wheat Improvement Center (CIMMYT) and the International Center for Agriculture Research in the Dry Areas (ICARDA), have successfully released over 100 bread wheat and more than 40 durum wheat varieties since the late 1960s. This impressive track record of variety release, which has been supported by several national and international investments, is considered to be an important factor behind the rapid wheat productivity gains reported in Ethiopia in recent years. In parallel there have been extensive efforts to fast-track the release of rust resistant varieties through favourable policy measures from the Ethiopian government, plus seed promotion and dissemination efforts from different research and development partners. However, reliably tracking varietal penetration and dynamics in farmers’ fields remains a challenging task.

Developing and diffusing improved varieties has been at the core of agricultural development for decades, being one of the most impactful agricultural technologies available to farmers. Varietal use studies provide a fundamental measure of the success and effectiveness of agricultural research and investment and an important source for learning. Yet obtaining accurate information on crop varieties diffusion is a challenging endeavour. Variety use studies conventionally used recall surveys (e.g. farm household surveys or expert opinion), but this is increasingly questioned as they are imperfect and an error-prone means to determine varietal identity and use. In the most recent and comprehensive effort to estimate the diffusion of improved varieties in sub-Saharan Africa^22^, it was concluded that “probably neither surveys nor expert panels can do a good job in delivering accurate estimates of cultivar-specific adoption”.

DNA fingerprinting was developed over three decades ago^23,24^ and has subsequently transformed human forensic diagnostics. However, only recently have efforts begun to explore its application to crop varietal identification and use. Rapidly decreasing per sample genotyping costs and increasingly powerful biometric analysis have now made DNA fingerprinting feasible to consider for use in mainstream applications for crop varietal use studies. DNA fingerprinting is considered to be a reliable method to accurately identify varieties grown by farmers and thereby increasingly seen as the “gold standard” for varietal identification. With reducing costs and potential applicability^25^, there is an increasing number of pilot studies looking at the technology for variety adoption studies^26–30^. Dreisigacker et al.^31^ successfully used DNA fingerprinting at scale to measure wheat varietal adoption in 4 provinces in Afghanistan around contrasting village hubs that had received, or not received, seed of improved varieties.

**Figure 1:**
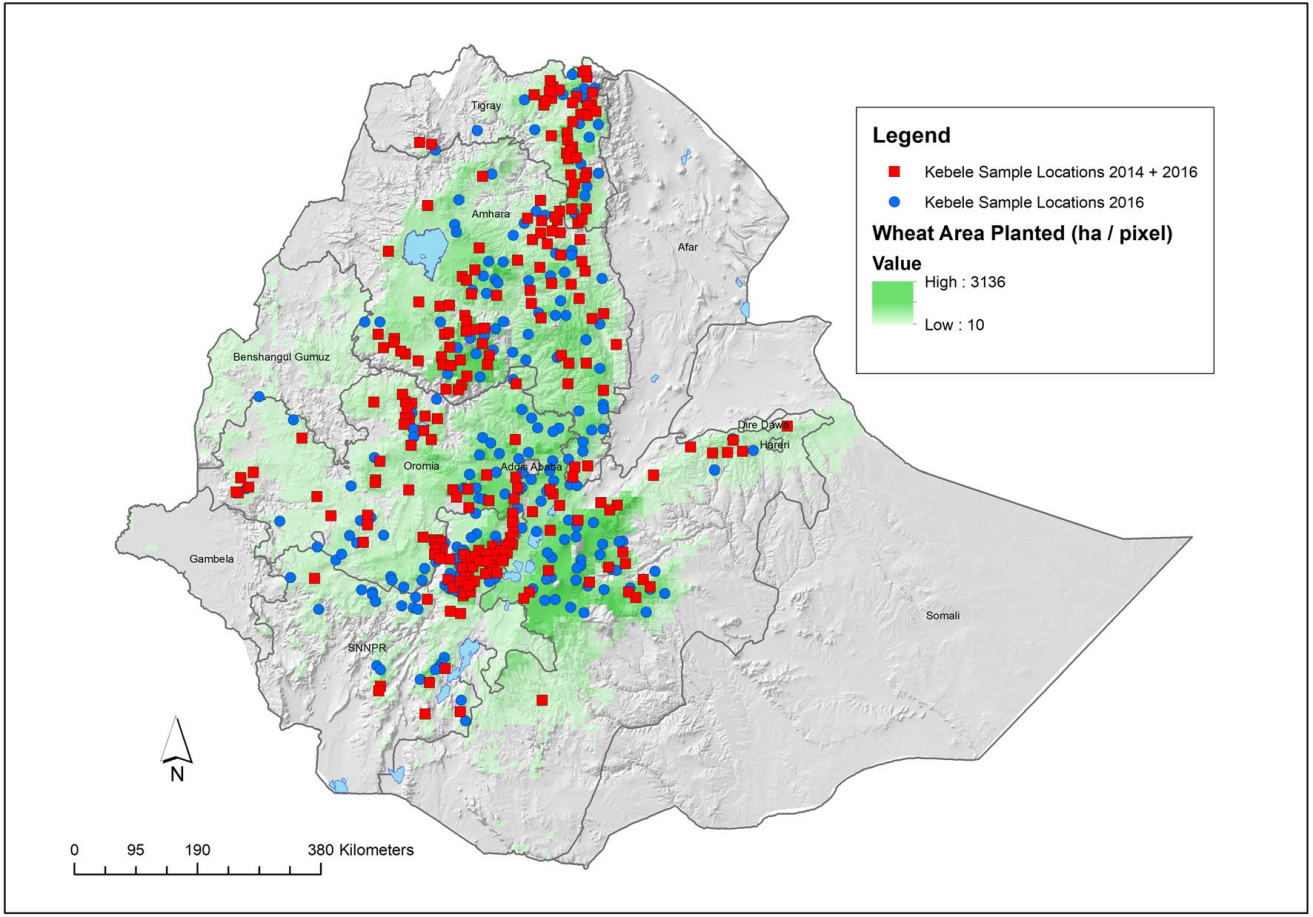
Location of sample kebeles (centroids) covered in the 2014/15 and 2016/17 wheat sample collections in relation to the wheat growing area (green). Red squares indicate Kebeles sampled in both 2014/15 and 2016/17 (n = 239). Blue dots indicate additional Kebeles sampled only in 2016/17 (n = 193) [map generated by DPH using ESRI ArcGIS 10.6 https://www.esri.com/en-us/arcgis/products/arcgis-pro/overview].

Several wheat adoption studies have been undertaken in Ethiopia over the last decade^11,32–34^ , but all have so far relied solely on farmers’ recall and/or expert opinion to estimate cultivar-specific use. The first large-scale application of DNA fingerprinting to assess wheat diffusion in Ethiopia collected data in 2016/17 (Fig. 1) and is reported here. This effort, to the best of our knowledge, is the first to look at a large, nationally representative sample of a staple crop and using DNA fingerprinting for variety identification. As such, it represents a milestone in variety use research and gives an accurate, objective diffusion estimate of improved wheat varieties at country scale.

## Materials and methods

### Wheat varieties released in Ethiopia and reference library development

Formal wheat breeding in Ethiopia started in the 1960s with the objective of developing high yielding varieties with resistance to major biotic and abiotic stresses. The breeding program had been receiving wheat germplasm from international and national research institutes such as CIMMYT, ICARDA and Kenya besides using wheat landraces collected within the country for variety development. The national and regional breeding programs released 133 bread and durum wheat varieties over a period of 50 years (1967–2016). For this study, a comprehensive wheat reference library was created by collecting 111 varieties out of the 133 total wheat varieties released in Ethiopia. Breeder seed for all reference varieties were obtained directly from the research centres that released the varieties or the national wheat coordinating research centre at Kulumsa. It was not possible to obtain seed for the missing 22 varieties—all of which were old and considered to be out of production. In most cases multiple collections of breeder seed were sampled for each variety and the resultant genotypic data analysed for consistent results across samples from the same variety.

### Field sampling

Field sampling and farmer surveys were undertaken by the Central Statistical Agency (CSA) of Ethiopia as part of the annual Agricultural Sample Survey (AgSS). This survey is the largest survey undertaken in Ethiopia with approximately 45,000 rural households surveyed each year. The AgSS includes all cereals, pulses and oilseeds. Crop cuts for yield are an integral part of the AgSS and hence provided the opportunity to obtain grain samples that could be sub-sampled for DNA fingerprinting. The AgSS also includes a farmer survey that covers basic farm household indicators (e.g. demographics, land size, input usage, crop choice and output marketing). For the DNA fingerprinting study, an additional supplemental survey instrument was also included. The AgSS and supplementary survey were carried out following all guidelines and regulations of the Ethiopian Government, with approval from the CSA Institutional Review Board that all required confidentiality and ethical standards for surveys were met. Voluntary, informed consent was obtained in all on-farm surveys prior to interview. CSA is mandated by law in Ethiopia as the official agency to conduct surveys, undertake collection of data, analyze and report findings at national and local level. The Enumerators are employees of CSA and they are approved and trained by the agency to conduct surveys as per the Agency’s guidelines, which take into account the laws of the land and approved by the Institutional Review Board. In this case, the enumerators are given the legal authority to collect information from farmers following the guidelines of the agency, one of which is getting the voluntary consent of each and every farmer involved in the survey. This being done orally in local languages to eliminate literacy issues following approved procedures. The enumerators live around the survey areas, are part of the local communities, and they are fully aware of both the national and local laws. Given this legal mandate and authorization we would consider them to be legally authorized representatives for the voluntary informed consent process. The informed consent process that was undertaken by CSA followed fully approved procedures, with the CSA Institutional Review Board approving the informed consent process that was undertaken. This is an integral part of the surveys and follows their procedures. All participating households/illiterate farmers provided full consent orally before proceeding with the interview, with the enumerator who is the Legally Authorized Representative signing to confirm that full informed consent had been given.

During the main (*Meher*) wheat growing season of 2016/17, a large, nationally representative wheat sample was collected by CSA under the framework of the AgSS. Sampling was undertaken in the four major wheat producing regions (Amhara, Oromia, Tigray and SNNPR [Southern Nations, Nationalities, and People's Region]—which collectively represent 92% of national wheat production) using the standard AgSS stratified two-stage cluster sample design. Enumeration Areas (EA’s) in each zone (based on the kebele—the lowest administration unit in Ethiopia), were randomly selected using a probability proportional to size sampling technique; with size being the number of agricultural households. For the DNA fingerprinting study in 2016/17, 432 EA’s were selected from the four wheat-growing regions—including 239 EA’s [kebeles] that were also sampled in 2014/15 using the same methodology (see Fig. 1). Subsequently, 20 agricultural households within each sample EA were randomly selected from household lists. Ten wheat plot crop cuts per EA (kebele) were then undertaken. The crop cuts were done on a randomly located 4 ×  4 m^2^ sub-plot within a randomly selected field. For the DNA sample, grain from the 4 ×  4 m^2^ crop cut was air dried to constant weight, mixed and a random 200–250 g sample of grain selected. A total of 3909 wheat field samples were collected in 2016/17, with genotypic data obtained from 3771 (and in 2014/15 a total of 1635 samples, 1586 genotyped). All field samples and surveys were barcoded to ensure accurate tracking and matching of results from field to final genotyping result.

This sampling methodology permitted a large, representative sample of the main wheat growing areas in Ethiopia to be obtained. It was particularly cost-effective as sampling and surveys were undertaken as part of the existing annual AgSS national survey.

All grain samples and filled-in survey questionnaires were transported to EIAR National Agricultural Biotechnology Research Centre (NABRC) at Holeta. DNA was successfully extracted from 3771 samples for the 2016/17 season at Holeta and genotyped using DArTseq at DArT in Canberra, Australia as described below.

### DNA fingerprinting

#### Sample processing

10 g of seed of each reference variety or 10 g of grain from field-collected sample was randomly sampled from each of the bags of seed/grain. The grain sample was milled for 30 s using a compact cross blade blender (High-speed Multi-function Comminutor: RRH-100) to produce a homogenous flour sample. The flour was cooled to room temperature and placed into two 50 ml glass, screw lid storage tubes and refrigerated at 4 °C until use. The blender was cleaned thoroughly between millings using 70% ethanol wipes. For the reference materials, 3 independent samples were taken from each bag to provide sub-replication in addition to the different varietal sources sampled.

#### DNA extraction

Wheat DNA was extracted from 100 mg sub samples of flour at NABRC using a modified DArT DNA extraction protocol with Zymo Kit^35^. Briefly, 100 mg of flour was transferred to 1.2 ml 8-strip micro tubes arranged in 96 well racks and 400ul of extraction buffer was added to each tube followed by a thorough mixing. Tubes were incubated at 65 °C for 60 min, chilled for 45 min at 4 °C, mixed with 400 µl of 4 °C 5 M NaCl and incubated on ice for a further 15 min. After centrifugation (6300 RPM for 10 min) to remove any debris, 200 µl of the supernatant was mixed with 600 µl of Zymo kit binding buffer. From each sample 150 µl of this solution was loaded into the specific well in a Zymo binding plate until 94 samples were loaded (wells G12 and H12 were left empty for genotyping controls). Two washes were performed using 200 μl of zymo wash buffer and 2 min of centrifugation at 2000 RPM according to Zymo kit recommendations and the final DNA was eluted with 50 µl of elution buffer. DNA quality and concentration were evaluated by gel electrophoresis on 0.8% agarose gels. DNA was stored at 4 °C.

#### DArTseq genotyping

DNA was shipped to Diversity Arrays Technology Pty Ltd laboratories in Canberra, Australia for processing using the DArTseq platform using protocol optimised for wheat and used to process hundreds of thousands of wheat accessions. All methods described were based on modified versions of those described by Kilian et al.^36^. The DArTseq method is highly standardised and therefore the same methods are described in numerous research papers, covering a wide range of topics^37,38^. DNA samples were processed in digestion/ligation reactions using a combination of PstI and HpaII Restriction Enzymes (RE), principally as per Kilian et al.^36^ but replacing a single PstI-compatible adaptor with two different adaptors corresponding to two different RE overhangs. The PstI-compatible adapter was designed to include Illumina flowcell attachment sequence, sequencing primer sequence and “staggered”, varying length barcode region, similar to the sequence reported by Elshire et al.^39^. Reverse adapter contained flowcell attachment region and HpaII-compatible overhang sequence.

Only “mixed fragments” (PstI-HpaII) were effectively amplified in 30 rounds of polymerase chain reaction (PCR) using the following reaction conditions: 94 °C for 1 min, 30 cycles of; 94 °C for 20 s, 58 °C for 30 s, 72 °C for 45 s, followed by a final hold of 72 °C for 7 min.

After PCR, equimolar amounts of amplification products from each sample of the 96-well microtiter plate were bulked and applied to c-Bot (Illumina) bridge PCR followed by sequencing on Illumina Hiseq2500. The sequencing (single read) was run for 77 cycles.

Sequences generated from each lane were processed using proprietary DArT analytical pipelines. In the primary pipeline the fastq files were first processed to filter away poor-quality sequences, applying more stringent selection criteria to the barcode region compared to the rest of the sequence. In that way the assignments of the sequences to specific samples carried in the “barcode split” step were very reliable.

Filtering was performed on the raw sequences using the following parameters:

- Barcode region: Min Phred pass score 30, Min pass percentage 75;
- Whole read: Min Phred pass score 10, Min pass percentage 50.

Approximately 2,000,000 sequences per sample were used in marker calling. Identical sequences were collapsed into “fastqcoll files” which were then “groomed” using DArT PL’s proprietary algorithm and used in DArT’s proprietary SNP calling pipeline DArTsoft14, with a threshold sequence distance of 3 basepairs (https://www.diversityarrays.com^38^) Clusters were parsed into separate SNP loci using a range of technical parameters, especially the balance of read counts for the allelic pairs. In addition, multiple samples were processed as technical replicates (from DNA to allelic calls) and scoring consistency was used as the main selection criteria for high quality/low error rate markers.

#### GeneticID/purity determination

DArTsoft14 pipeline outputs were used in a second DArT PL pipeline for genetic identity and purity definition. This pipeline is based on a heuristic, data-driven approach geared to provide the provides most likely variety match along with a clear indication of any potential ambiguity arising from reference varieties samples which are not genotypically identical.

The pipeline starts with grouping (binning) the DNA profiles obtained from varietal reference samples based on their genetic distances to form Varietal Bins (VBs).

##### Varietal bin formation

Two complementary distance measures are applied as both provide with somewhat different groupings. The first method, commonly applied to the binary and discrete data, the Hamming Distance (HD), uses Single Nucleotide Polymorphism (SNP) calls based on presence/absence of one (or both) alternative alleles (forms) of the SNP marker in a particular sample assay. In the next step a hierarchical clustering (single linkage) algorithm is applied to split samples into groups based on SNP profile similarity. In parallel, the quantitative sequence data (counts for the sequences with allelic variants of all SNPs identified as polymorphic among varieties included in analysis) are used to calculate the Mahalanobis Distance (MD). MD is the wildly used distance measure for quantitative (especially highly correlated) traits in classification applications and outperformed in our tests other distance measures, including commonly used Euclidean Distance. MD matrix is then subjected to the same clustering algorithm as the HD and the two sets of resulting bins are merged to form VBs.

In the majority of cases the VBs are discrete, representing one variety. However, in some cases, varietal references cannot be separated from one another and a merged VB is formed comprising two or more varieties. In this case, in addition to forming the VBs the pipeline searches for the SNPs and markers that are unique to or diagnostic of a particular varietal reference within the multi-variety VB.

In cases when the pipeline detects inconsistency of profiles among the multiple shipments of the same named variety the search for private alleles is restricted to one of the submissions, but input files for analysis can be created to search for markers separating same named varieties or any user-defined grouping of those.

The Hamming distances are than calculated between all varieties within a bin (if the bin is “multivarietal”) using diagnostic markers only. The resulting distance matrices are than subject again to clustering analysis using the same single linkage algorithm and Sub-bin references are created. This step concludes the creation of reference profiles and enables testing of the field/test samples against these references.

##### Testing farmer collected samples

Each test sample profile is compared against the panel of reference profiles and the top scoring variety is identified using a “matching index”, which takes into account the proportion of alleles present in the reference which were also found in the test sample AND the proportion of “foreign” alleles, which were not detected in the reference profile being matched. At this step the “purity” is calculated, which represents the fraction of alleles in the sample that are “foreign” to the matched reference bin. In the next step the pipeline checks if the best scoring reference belonged to a VB with one or more varieties. If the match is to a VB with a single reference variety the identity of the test sample is declared as this variety. If the match is to a member of a larger multi-variety VB the test sample is compared against ALL members of this reference bin using diagnostic markers for the sub-bins and the variety with the highest level of similarity is declared as the most likely identity of the rest of the sample.

### Phenotypic varietal verification of unidentified samples

DNA fingerprinting could not identify 228 samples out of 3771 (6%) in the 2016/17 season—i.e., there was no accurate match of the sample to any material in the reference library. Screen house grow out plots were conducted at the EIAR Debre Zeit research centre using residual seed of these unclassified DNA samples. Phenotypic evaluation revealed that the majority of these samples were actually Triticale (n = 164)—a hybrid of wheat and rye. Triticale varieties were not included in the original reference set used for DNA fingerprinting and hence could not be classified. The remaining unidentified samples included some bread wheat (n = 43), a small number of durum wheat (n = 7) and mixed samples (bread + durum + triticale, n = 4). These unidentified bread wheat and durum wheat samples were assumed to either not be represented varietally in the reference set and/or to have been subject to varietal mixing and/or genetic drift over time resulting in non-classification.

## Results

### Varietal use—DNA fingerprinting vs farmers’ perspective

**Figure 2:**
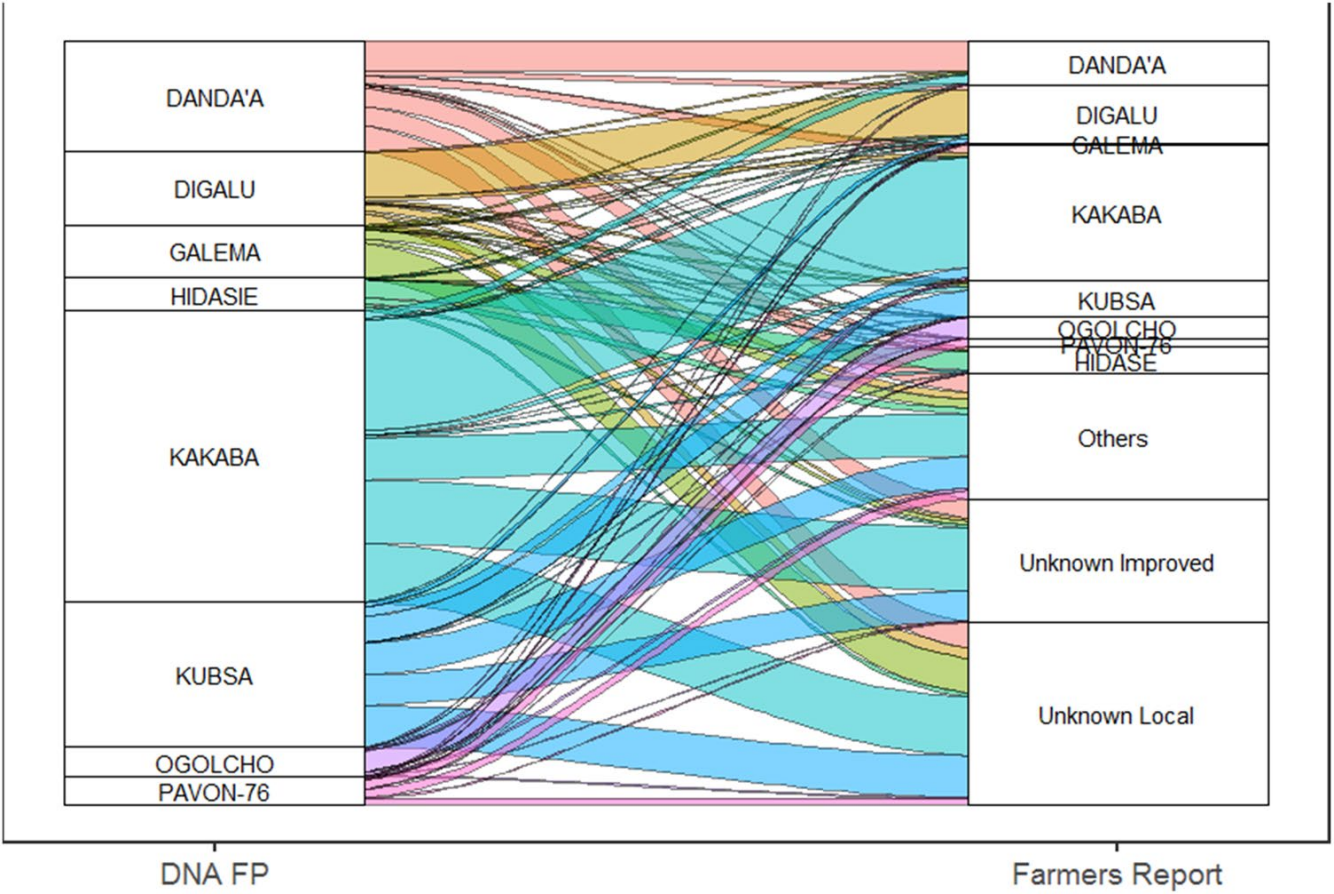
Sankey diagram illustrating the relationship between widely grown wheat varieties identified by DNA fingerprinting (left) and corresponding wheat variety names given by farmers (right) for the same plot. Box height indicates the percentage of total varieties, while lines illustrate the relationship [created by MJ using R 4.0.2 https://www.r-graph-gallery.com/sankey-diagram.html].

From the large-scale, crop-cut grain sampling undertaken in 2016/17 for genotyping (n = 3771 genotyped samples/plots), DNA fingerprinting identified 3543 samples (94%), including 45 unique wheat varieties—38 bread and 7 durum wheat varieties from the panel of 111 references. In contrast to this, farmer reports named 56 varieties from the same sample plots—51 bread wheat and 5 durum wheat varieties (SI Figure 2). There was a large degree of mismatch between farmer reports and DNA fingerprinting results, with only 28% of farmers (n = 989 out of 3543 samples identified using DNA fingerprinting) able to provide a name for the variety they have grown that exactly matched the corresponding DNA result from the same plot. Mismatches for eight widely grown varieties are illustrated in Fig. 2. Ambiguity was also much higher using farmer recall with 49% (1917 out of 3880 farmer reports) classed as “unimproved/local”, “improved” or “unknown”—i.e. not assigned to any known variety. A large proportion of the farmer reports were either “local” or “unimproved” (n = 1233; 32% of reports), but DNA results generally did not support this farmer classification. Based on DNA data, the majority of these farmers were actually growing released improved varieties, albeit of varying age post release. The bulk of unidentified samples from DNA fingerprinting (4% of 3771 genotyped samples) were actually Triticale and not wheat. The DNA fingerprinting could thus potentially be made even more accurate by expanding the reference library, possibly including wheat related species such as Triticale where relevant. Overall, from the results obtained there was low confidence in the farmer reports providing reliable estimates of actual wheat variety use in Ethiopia in 2016/17.

### Varietal diffusion—DNA fingerprinting vs. farmers’ perspective

**Figure 3:**
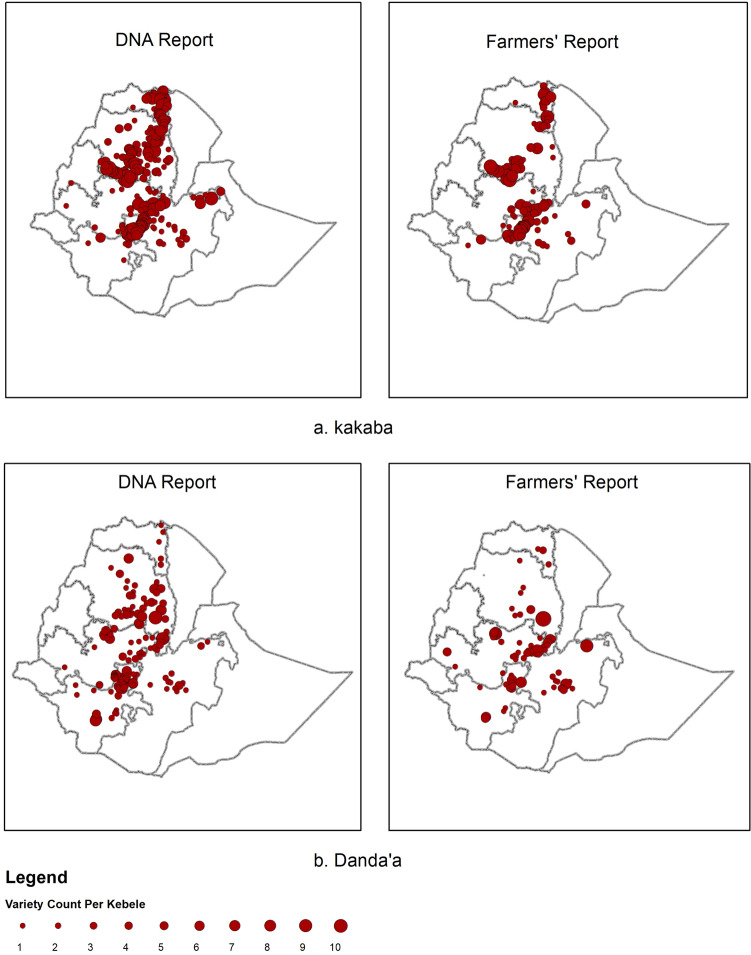
Geographical distribution of variety *Kakaba* (**a**) and *Danda’a* (**b**) using DNA fingerprinting data (left panels) and farmer reports (right panels; 2016/17, 432 kebeles. Size of dots represent the number of plots per Kebele) [maps generated by YA using ESRI ArcGIS 10.6 https://www.esri.com/en-us/arcgis/products/arcgis-pro/overview].

DNA fingerprinting revealed that farmers have widely adopted recently released, rust resistant wheat varieties at scale. The varieties ‘*Kakaba’* and ‘*Danda’a’*, both adult plant stem rust resistant varieties, released in 2010, serve as prominent examples. ‘*Kakaba’* was the most frequent variety identified by DNA fingerprinting in 2016/17 (27% of all samples) and was distributed across all the main wheat growing areas of Ethiopia (Fig. 3a). Similarly, ‘*Danda’a’* the third most frequent variety identified by DNA fingerprinting (10% of all samples) was being grown widely across Ethiopia (Fig. 3b). Reliance solely on farmer reported distributions for these varieties would have resulted in considerable under reporting, most noticeably in Amhara region.

**Figure 4:**
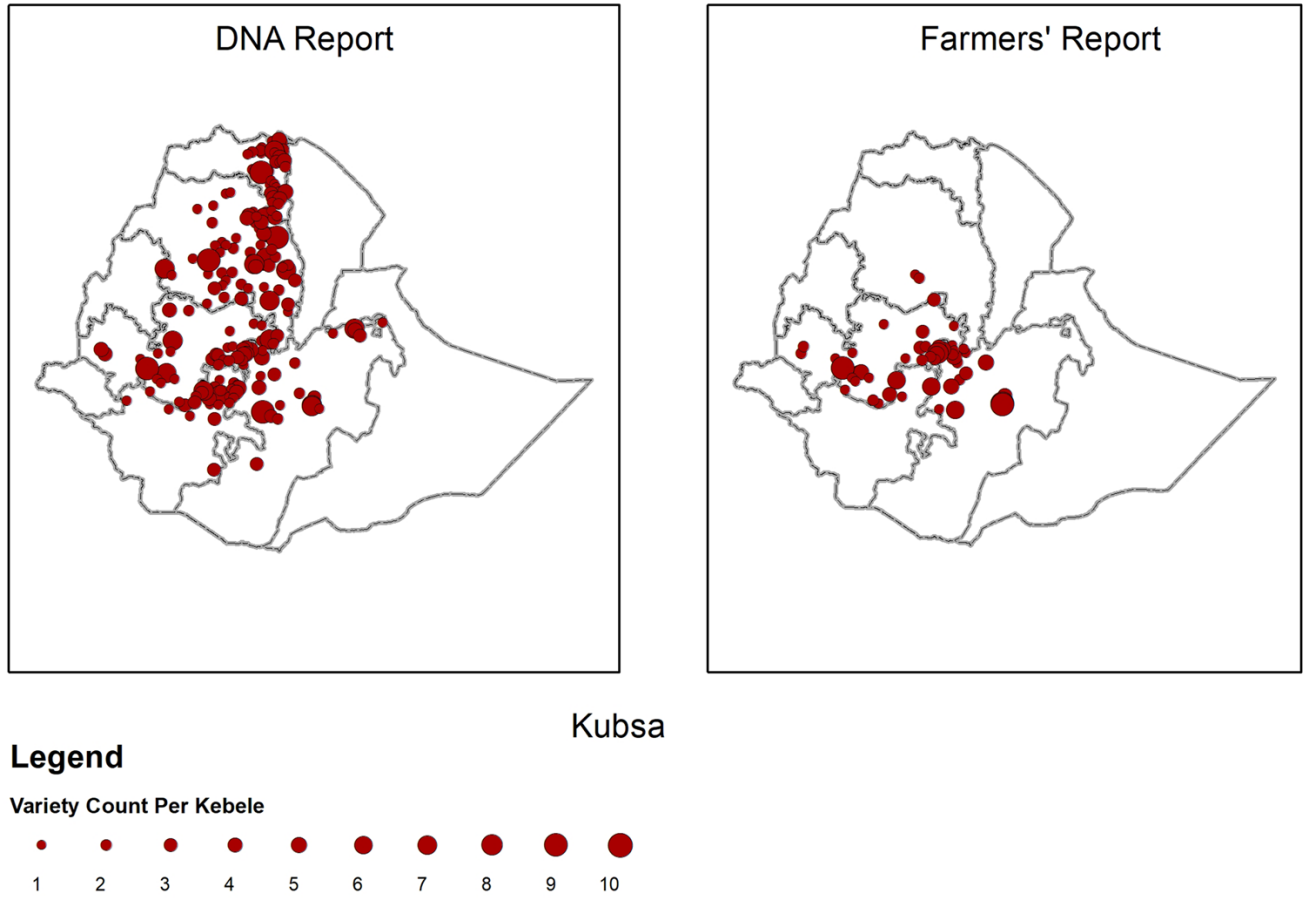
Geographical distribution of variety *Kubsa* variety using DNA fingerprinting (left panel) and farmers reports (right panel; 2016/17, 432 kebeles. Size of dots represent the number of plots per Kebele) [maps generated by YA using ESRI ArcGIS 10.6 https://www.esri.com/en-us/arcgis/products/arcgis-pro/overview].

**Figure 5:**
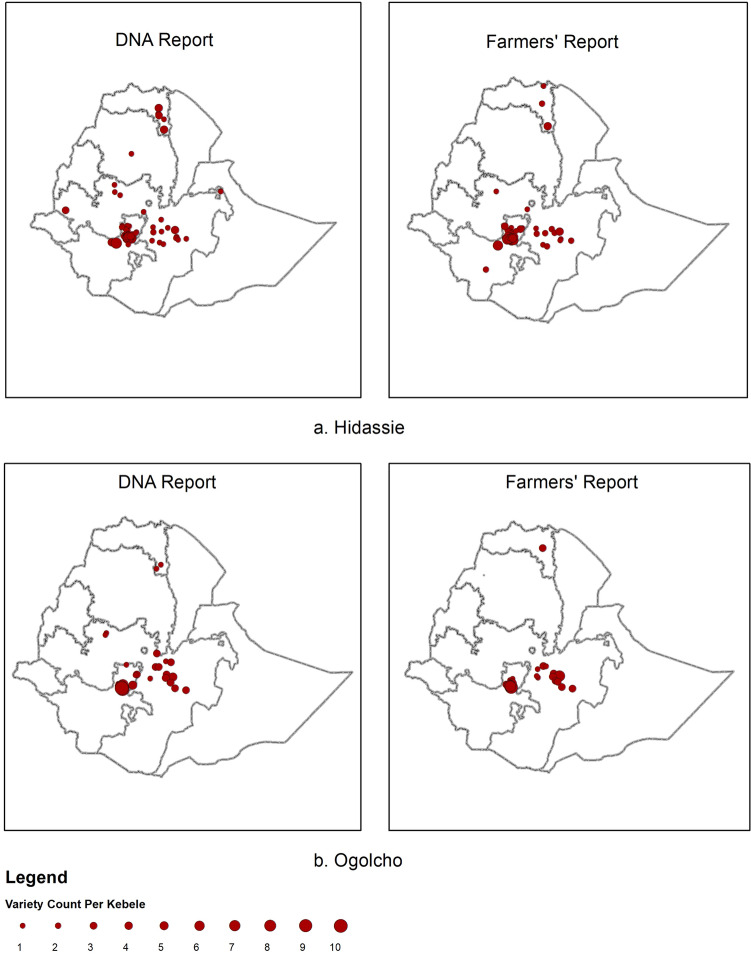
Geographical distribution of variety *Hidasie* (**a**) and *Ogolcho* (**b**) using DNA fingerprinting data (left panels) and farmer reports (right panels; 2016/17, 432 kebeles. Size of dots represent the number of plots per Kebele) [maps generated by YA using ESRI ArcGIS 10.6 https://www.esri.com/en-us/arcgis/products/arcgis-pro/overview].

The observed disparity in farmer reported variety distributions compared with DNA-based identification was greatest for older varieties. The variety ‘*Kubsa’*, released in 1995, was until recently considered to be the most widely grown wheat variety in Ethiopia due to wide adaptability, food quality, and marketability; factors which led to strong farmer preference^40^. High susceptibility to new variants of stripe rust in 2010 is considered to have resulted in some dis-adoption of ‘*Kubsa’* by farmers^15,33,34^. DNA fingerprinting revealed that ‘*Kubsa’* was still widely grown across Ethiopia, being the second most frequently identified variety (13% of all samples). In contrast, ‘*Kubsa’* was reported by only a small proportion of farmers (Fig. 4) reflecting a substantial discrepancy in spatial distribution compared with the DNA data. Conversely, for newly released varieties exemplified by ‘*Hidasie’* and ‘*Ogolcho’* (both stem rust resistant varieties released in 2012) there was relatively close spatial alignment between DNA fingerprinting results and farmer reports (Fig. 5). Additional variety spatial distributions based on DNA fingerprinting results and farmer reports are given in the SI (SI Figs. 2–6).

### Varietal prevalence and turnover

The top 10 most commonly grown varieties identified by DNA fingerprinting occupied 81.2% of the area sampled in 2016/17 (Table 1). Four of these top 10 most widely grown cultivars (*Kakaba, Danda’a, Hidasie, Ogolocho*) were recently released rust resistant varieties developed through the EIAR and CIMMYT breeding programmes. Nearly half (47.3%) of the 2016/17 area sampled was grown to varieties not older than 10 years; whereas within this group, varieties released after 2005 accounted for 61%. About a quarter (27.3%) of area sampled was covered by varieties older than 20 years. The persistence of older varieties was most pronounced for durum wheat.

**Table 1:** Wheat varietal use indicators for 2016/17 (identified by DNA fingerprinting; 432 kebeles) wheat varieties.

Variety	Year released	Wheat area		
		Area(ha)	Individual %	Cumulative %
Kakaba	2010	183.1	27.1	27.1
Kubsa	1995	86.6	12.8	39.9
Digalu	2005	69.7	10.3	50.3
Danda'a	2010	65.7	9.7	60.0
Galema	1995	39.2	5.8	65.8
(Bobicho /Senkegna)^a^	2002	35.0	5.2	71.0
Pavon-76	1982	24.2	3.6	74.6
Hidasie	2012	20.1	3.0	77.5
Ogolcho	2012	18.8	2.8	80.3
Arendato (durum)	1967	15.0	2.2	82.5
Hawi	1999	13.6	2.0	84.6
Simba	1999	13.1	1.9	86.5
Tussie	1997	11.3	1.7	88.2
Huluka	2012	7.8	1.2	89.3
Sirbo	2001	7.4	1.1	90.4
Mada Walabu	1999	6.9	1.0	91.5
Lasta (durum)	2002	5.8	0.9	92.3
Bolo	2009	5.6	0.8	93.2
Others (22 bread and 5 durum varieties)	1967–2016	46.2	6.8	100.0
Total		674.9		

^a^Uncertainty exists over the breeder seed source obtained for this variety. Two very closely related varieties could not be separated^49^.

The cross wheat species area weighted average age of varieties^41^ was 14 years (based on DNA sampling in 2016/17 and all wheat varieties). Analysis of bread and durum wheat varieties separately revealed a marked disparity; with the average varietal weighted age for bread wheat being 12.8 years, compared to 39.1 years for durum wheat.

Repeat DNA sampling in 239 kebeles from two different seasons, 2014/15 and 2016/17, revealed some insights into spatial dynamics over a short 2-year period. Table 2 lists the predominant varieties and the number of kebeles growing these in 2014/15 and 2016/17. Varieties diffusing into new kebeles were all recent releases with rust resistance. Conversely, varieties with a decreasing footprint in terms of kebeles (indicating possible dis-adoption) were older releases with current susceptibility to wheat rusts.

**Table 2:** Use and spatial diffusion indicators of selected wheat varieties (identified by DNA fingerprinting; only for the 239 kebele’s included in both 2014/15 and 2016/17).

Variety	Year released	Kebeles growing (#)			Comment
		2014/15	2016/17	Change	
**Kakaba**	2010	75	144	** + 69**	
Kubsa	1994	137	104	− 33	Stripe rust susceptible (2010)
**Danda’a**	2010	22	68	** + 46**	
Digalu	2005	50	48	− 2	Rust susceptible—stem (2013), stripe (2016)
**(Bobicho/Senkegna)**	2002	10	64	** + 54**	Uncertainty exists over breeder seed source
Galema	1995	47	33	− 14	Stripe rust susceptible (2010)
**Hidasie**	2012	0	27	** + 27**	
**Ogolcho**	2012	2	13	** + 11**	
Arendato	1967	30	22	− 8	Durum wheat
Pavon-76	1982	23	20	− 3	

Varieties in bold are those showing diffusion into new kebeles in 2016/17.

### Durum wheat identification

Nationally representative sampling of wheat plots in 2016/17 resulted in only a very small proportion of durum wheat being detected with DNA fingerprinting: only 3% (119 out of 3,771 genotyped samples/plots), representing just 4% of the area sampled. An even smaller number of durum wheat plots were reported by farmers—0.5% (22 plots out of 3880 farmer reports). DNA analysis identified seven durum wheat varieties, with 11 samples comprising unidentified durum’s. Farmers reported growing five durum varieties, but there was little correspondence between DNA results and farmer reports (SI Table 1).

DNA data indicated that the very old variety *Arendato* (1967 release, a selection from Ethiopian landrace “Arendato”) was the most frequent durum in the sampled plots (Arendato was the close genetic match, but it is possible that other genetically similar landraces were sampled). There was little evidence of recent durum varieties being grown in the sampled areas. Most durum wheat plots detected by DNA fingerprinting were located in the eastern highlands of Amhara and Tigray.

### Germplasm sources for varieties in use

**Figure 6:**
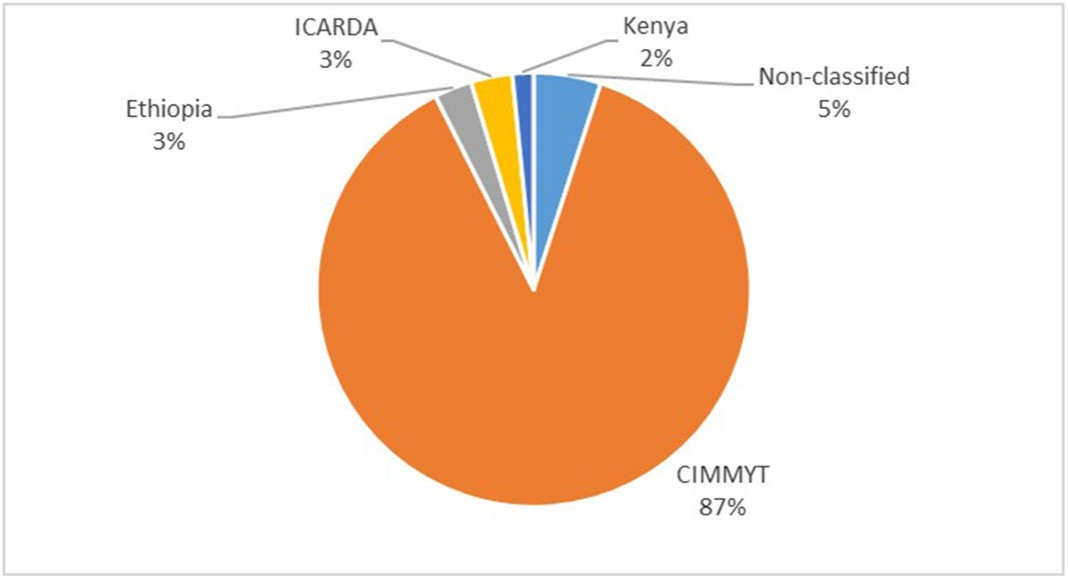
Ethiopia’s wheat area by germplasm sources (identified by DNA fingerprinting; 2016/17 season, 432 kebeles) [created using MS Excel 2016 by MJ https://www.microsoft.com/en-gb/microsoft-365/excel] (non-classified are varieties not identified by DNA sequencing).

From the 3543 wheat plots with identified varieties through DNA Fingerprinting in 2016/17, 3236 (91%) of the plots had varieties developed using germplasm originating from CIMMYT, 122 (4%) from Ethiopian germplasm, 112 (3%) from ICARDA germplasm and 72 (2%) from Kenyan germplasm. In terms of wheat area, varieties developed using germplasm received from CIMMYT and ICARDA respectively cover 87% and 3% of the wheat area surveyed in 2016/17 (Fig. 6, including unidentified). Clearly CGIAR germplasm, notably CIMMYT-derived material, has made a significant contribution to the wheat varieties being grown in Ethiopia in 2016/17.

### Estimated impacts of recently released varieties

Due to recurrent wheat rust outbreaks in Ethiopia, both government and non-government organizations supported the multiplication, fast-track registration and dissemination of recently released rust resistant wheat varieties. Considering varieties released since 2005, DNA fingerprinting data shows that these recent varieties cover 61% of the sampled wheat area. On average, wheat varieties released since 2005 contributed to a yield increase of 220 kg/ha compared with older varieties based on all crop cut data. With a strong assumption that our sample plots are nationally representative, the average yield increment of these varieties contributed to an estimated production gain of 225,563 metric tons (t) during the 2016/17 main cropping season. Using a farm-gate price of 5000 Birr/t (approximately 221 US$ /t at the 2016/17 average exchange rate), these recently released wheat varieties contributed to an estimated income gain of US$ 49.8 M to wheat growing farmers. On the other hand, if these varieties had not been introduced and used by farmers, the government would have had to spend an additional US$ 50.8 M in 2016/17 to maintain the same level of national wheat consumption (based on 2016/17 wheat import price of US$225/t).

## Discussion

Improved crop varieties are amongst the most impactful agricultural technologies available to farmers. For decades crop improvement programs have been at the heart of agricultural development, providing farmers with higher yielding, (a) biotic stress tolerant and sometimes nutritionally enhanced varieties needed to feed growing populations. Adoption studies provide a fundamental measure of the success and effectiveness of agricultural research and investment and an important source for learning. Despite this critical nature, obtaining accurate information on the diffusion of crop varieties remains a challenging endeavour.

This study set out to reliably estimate wheat variety use in farmer’s fields in Ethiopia during 2016/17 and give an indication of variety turnover since 2014/15. Using large-scale, national crop cut surveys and the AgSS survey as a sampling frame it was possible to benchmark DNA fingerprinting results against conventional estimates (farmer reports from household surveys). Based on a comprehensive reference library, DNA fingerprinting was able to uniquely and accurately identify to the variety level 94% of the genotyped samples/plots. This contrasted with farmer reports only assigning a unique and recognised variety name to 49% of their survey plots. Direct comparison of DNA results with farmer reports for the same plots revealed that only 28% of farmers could give an accurate matching variety name. These results imply that variety use studies reliant solely on farmer recall are likely to be unreliable and potentially misleading. Jaleta et al.^42^ in a related paper, provide a detailed investigation on associated factors linked to farmer variety misidentification in Ethiopia and their implications. Consequently, adoption estimates may be incorrect and estimated impacts based on these adoption estimates could be questionable leading to misleading policy implications^26,43^. Whilst DNA fingerprinting provides more accurate varietal identification, it does not identify contextual and explanatory factors; hence, a combination of household survey and DNA fingerprinting approaches is recommended for reliable varietal adoption and impact assessments^42^.

DNA-based variety adoption data provided strong evidence that farmers in Ethiopia had widely adopted new, improved rust resistant varieties released by the Ethiopian wheat breeding programs. Ten varieties accounted for over 81% of the wheat area sampled and of these four were recently released (post-2010) rust resistant varieties. Recent releases, notably ‘*Kakaba’* and ‘*Danda’a’*, were distributed widely throughout the wheat growing areas of Ethiopia. In addition, time series DNA data collected from a repeated subset of kebeles in 2014/15 and 2016/17 gave a clear signal that recent, rust resistant wheat varieties were rapidly diffusing in contrast to older, largely rust susceptible wheat varieties which had contracting or static distributions. DNA time series data indicates that within just 4 years of release varieties can gain farmer acceptance and diffuse at scale (e.g., ‘*Hidasie’* and ‘*Ogolocho’* varieties). This dynamism in varietal adoption is considered to be driven by rapidly changing wheat rust pathogen populations, in combination with enabling policies and seed systems. Damaging rust epidemics, e.g., stripe rust in 2010 and stem rust in 2013, have caused farmers to seek alternative resistant varieties^20,34^. In combination, pro-active policies in the seed sector such as fast-track variety release, pre-release seed multiplication of rust resistant candidate varieties e.g., Danda’a and Kakaba and rust awareness campaigns by the EIAR and Ministry of Agriculture have also contributed^17,44^. The support and capacity building under different projects has played a critical role (e.g., Seed scaling supported by USAID, breeding for rust resistance supported by BMGF & DFID, and capacity building on breeding by World Bank through EAAPP). The strong partnership of CGIAR (CIMMYT and ICARDA on wheat) with the national agricultural research system has also played a pivotal role^45^. The success in speeding up variety release and seed multiplication in Ethiopia is considered a model for other countries.

Nonetheless, old varieties also persisted and continued to be grown by farmers in Ethiopia in 2016/17. This may inter alia imply that these varieties possess highly desirable traits preferred by farmers, consumers or processors; or that there is insufficient awareness of and/or access to seed of the new improved varieties. In reality, a combination of these could apply in different circumstances^15,40,46^. For the variety ‘*Kubsa’*, there is little doubt that some farmers still value the characteristics of this variety despite it being highly susceptible to stripe rust since 2010. Only DNA-based data revealed the true extent that ‘*Kubsa’* was still being grown in Ethiopia, with farmers either deliberately or unknowingly under reporting its use. Accurate information of this kind is extremely valuable to inform national decision-making and assessments regarding vulnerability and control strategies to prevent wheat rust epidemics.

DNA-based information also provided valuable insights on the likely status of durum wheat in Ethiopia. No official statistics are collected on the area and importance of durum wheat relative to bread wheat. Expert surveys since the 1980s have consistently indicated the rising popularity of bread wheat and the corresponding decline of durum wheat^9^. A significant shift thereby has occurred in Ethiopia’s wheat landscape over the last 30 years, with traditional tetraploid landraces being replaced with improved bread wheat varieties. Data from this study re-enforces and quantifies this notion, with durum wheat accounting for only 4% of the sampled area. The majority of durum wheat samples were an extremely old variety—*Arendato*; with no indication of diffusion of any of the newly released durum wheat varieties. This situation reflects the current limited, or even lack of, effective seed systems for durum wheat varieties in Ethiopia and the subsequent very limited adoption of new, improved varieties. Some initiatives are now trying to address and promote durum wheat^47^, but more work should be done to address the durum value chain in Ethiopia. Results of this study may potentially underreport the true status of durum wheat. For example if our sample underrepresented certain durum segments i.e. cultivation in isolated and remote pocket areas; durum landraces being grown on residual moisture outside of the main wheat growing seasons; or durum being grown more widely in the short rain (*Belg*) season, not covered in this study. Nonetheless, this study questions the extent of current cultivation of durum wheat in Ethiopia.

International wheat improvement programs from CGIAR centers have long-standing partnerships with the Ethiopian national wheat system. Germplasm in international nurseries, requested by national wheat program partners, have been evaluated and tested in Ethiopia for decades. This germplasm is considered to have played a major role in subsequent variety releases and productivity gains. Lantican et al.^48^ considered that CGIAR-related varieties contributed to 73% of all wheat varietal releases in sub-Saharan Africa, but no Ethiopia specific contribution was reported. This study confirmed the substantial contribution of CGIAR germplasm to varieties being grown by farmers in Ethiopia, with 94% of the plots and 90% of the area sampled contained wheat varieties derived from CGIAR germplasm.

The impacts of using new improved bread wheat varieties has been substantial. Initial economic benefit estimates put the gain to farmers, from varieties released after 2005, to be an additional 225,500 ton of extra production in 2016/17, a benefit valued at US$ 50 M. Widespread adoption of these improved bread wheat varieties, demonstrated by DNA fingerprinting, has clearly had a positive impact on both economic returns and national wheat production gains. Investments in wheat improvement made by international donor agencies, notably BMGF, DFID, World Bank, USAID and the Ethiopian government are thus resulting in a demonstrable positive impact in the wheat fields of Ethiopia. Innovative policy decisions relating to promotion, seed distribution and fast track release of varieties with characteristics desired by farmers, notably rust resistance, have resulted in new, improved wheat varieties rapidly being grown at scale in Ethiopia.

Despite the measured progress, challenges still remain, especially from the continually evolving threat posed by virulent new rust races and the influences of a changing climate. Continued investments in wheat improvement in Ethiopia will be required to maintain and increase wheat productivity in the future. This study has shown that DNA fingerprinting for varietal use tracking is feasible at large scale on important staple crops. Accurate assessment of the outcomes and impact of crop improvement programs in the future is likely to be increasingly dependent on the application of DNA fingerprinting technology.

## Supplementary information

Supplementary Information.

## Data Availability

Anonymous data used in producing the tables and figures are now shared in the public domain on the CIMMYT Dataverse repository; https://hdl.handle.net/11529/10548514.
