# Author Correction: Ethiopia’s transforming wheat landscape: tracking variety use through DNA fingerprinting

**DOI:** 10.1038/s41598-023-29767-7

**Published:** 2023-02-14

**Authors:** D. P. Hodson, M. Jaleta, K. Tesfaye, C. Yirga, H. Beyene, A. Kilian, J. Carling, T. Disasa, S. K. Alemu, T. Daba, A. Misganaw, K. Negisho, Y. Alemayehu, A. Badebo, B. Abeyo, O. Erenstein

**Affiliations:** 1 International Maize and Wheat Improvement Center (CIMMYT), Mexico City, Mexico; 2 International Maize and Wheat Improvement Center (CIMMYT), Addis Ababa, Ethiopia; 3 Ethiopian Institute of Agricultural Research (EIAR), Addis Ababa, Ethiopia; 4 Ethiopian Institute of Agricultural Research (EIAR), Holeta, Ethiopia; 5 Ethiopian Central Statistical Agency (CSA), Addis Ababa, Ethiopia; 6 Diversity Array Technologies (DArT), Canberra, Australia

Correction to: *Scientific Reports* 10.1038/s41598-020-75181-8, published online 28 October 2020

Abebaw Misganaw and Kefyalew Negisho were omitted from the author list in the original version of this Article.

The Author Contributions section now reads:

“D.P.H., M.J., K.T., C.Y., H.B., A.K. and O.E. designed the project and prepared the manuscript. H.B., C.Y. and M.J. planned and collected field data. A.K., C.J., T.D., S.K.A., A.M. and K.N. undertook lab analysis. D.P.H., M.J., K.T., Y.A., C.Y., A.K., and J.C. processed and analyzed the data. A.B., B.A., D.H. conducted phenotypic varietal verification of unidentified samples. All authors contributed to editing and correcting the manuscript.”

The original Article and accompanying Supplementary Information file have been corrected.

