# Supplementary material for: Ethiopia’s transforming wheat landscape: tracking variety use through DNA fingerprinting
Source: Sci Rep. 2020 Oct 28;10:18532. doi: 10.1038/s41598-020-75181-8 (PMC7595036; doi:10.1038/s41598-020-75181-8)
Supplement: Supplementary file 1 — Supplementary Information. [file 41598_2020_75181_MOESM1_ESM.docx]

**Supplementary Information: Ethiopia’s Transforming Wheat Landscape: Tracking Variety Use through DNA Fingerprinting**

Hodson, D.P.^1^, Jaleta, M.^2^, Tesfaye, K.^2^, Yirga, C.^3^, Beyene, H.^5^, Kilian, A.^6^, Carling, J.^6^, Disasa, T.^4^, Alemu, S.K.^4^, Daba, T.^3^, Misganaw, A.^3^, Negisho, K.^3^, Alemayehu, Y.^2^, Badebo, A.^2^, Abeyo, B.^2^, Erenstein, O.E.^1^

SI Figure 1. Wheat area, production and yield, 1960–2016, Ethiopia. Sources: FAO (2018). [Figure generated by DPH using MS Excel 2016 https://www.microsoft.com/en-gb/microsoft-365/excel]

1. DNA Fingerprinting


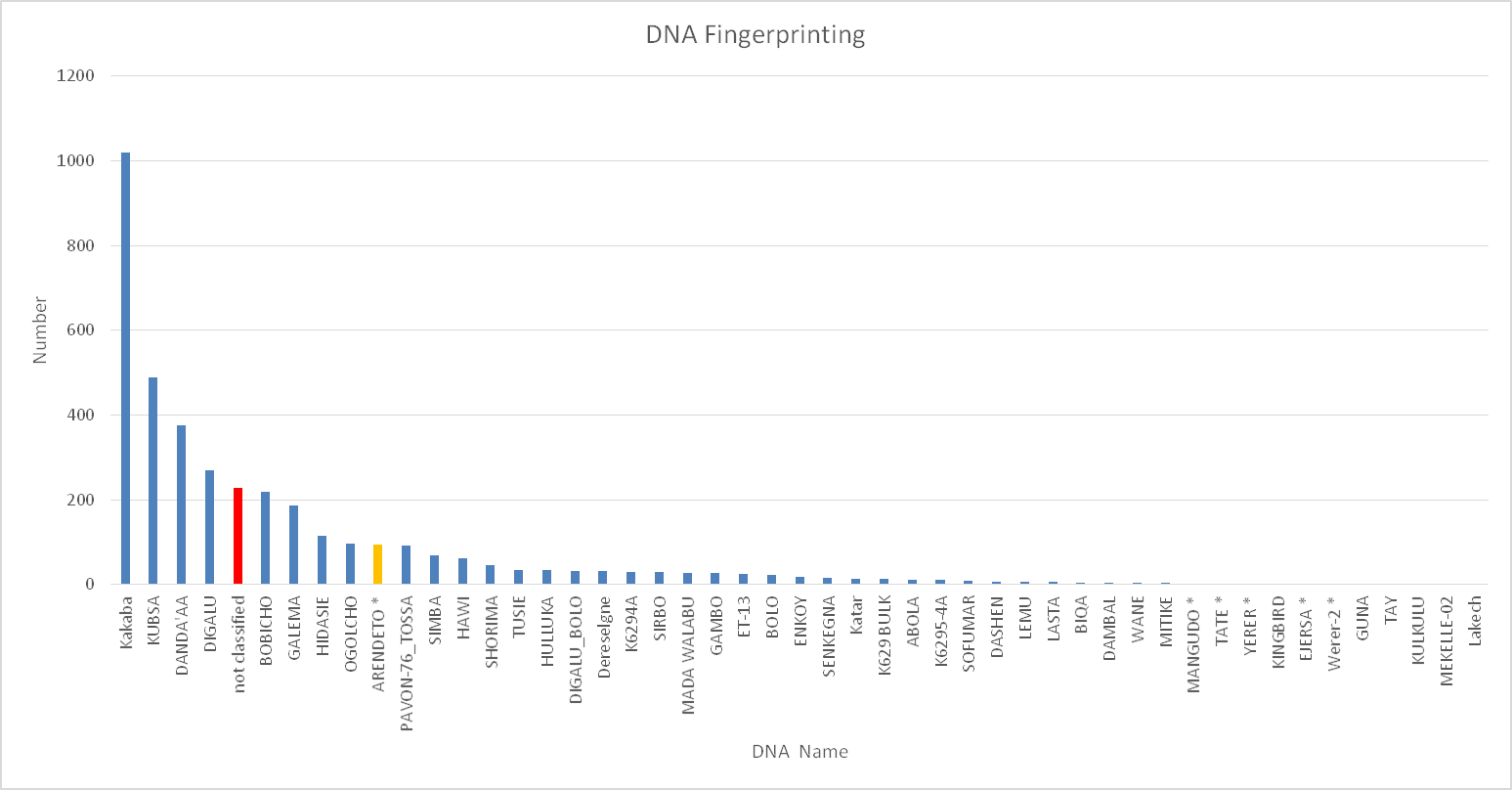


1. Farmer Reports


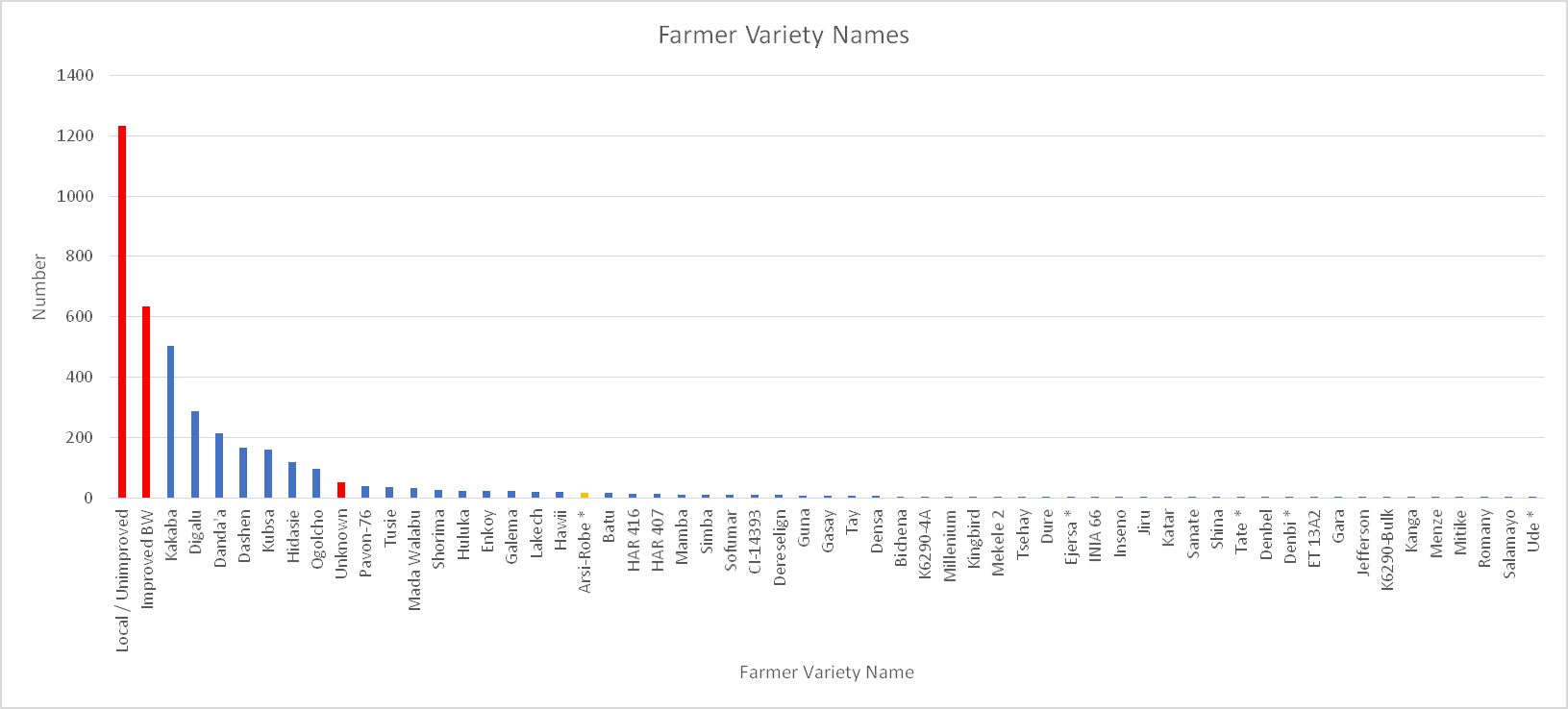


SI Figure 2. Wheat varieties identified by (A) DNA fingerprinting and (B) farmer reports ranked by detection frequency (2016/17, 432 kebeles; Red bars represent unclassified samples, blue bars/name represent bread wheat varieties, orange bars/name* represent durum wheat varieties) [Figure generated by DPH using MS Excel 2016 https://www.microsoft.com/en-gb/microsoft-365/excel]

**
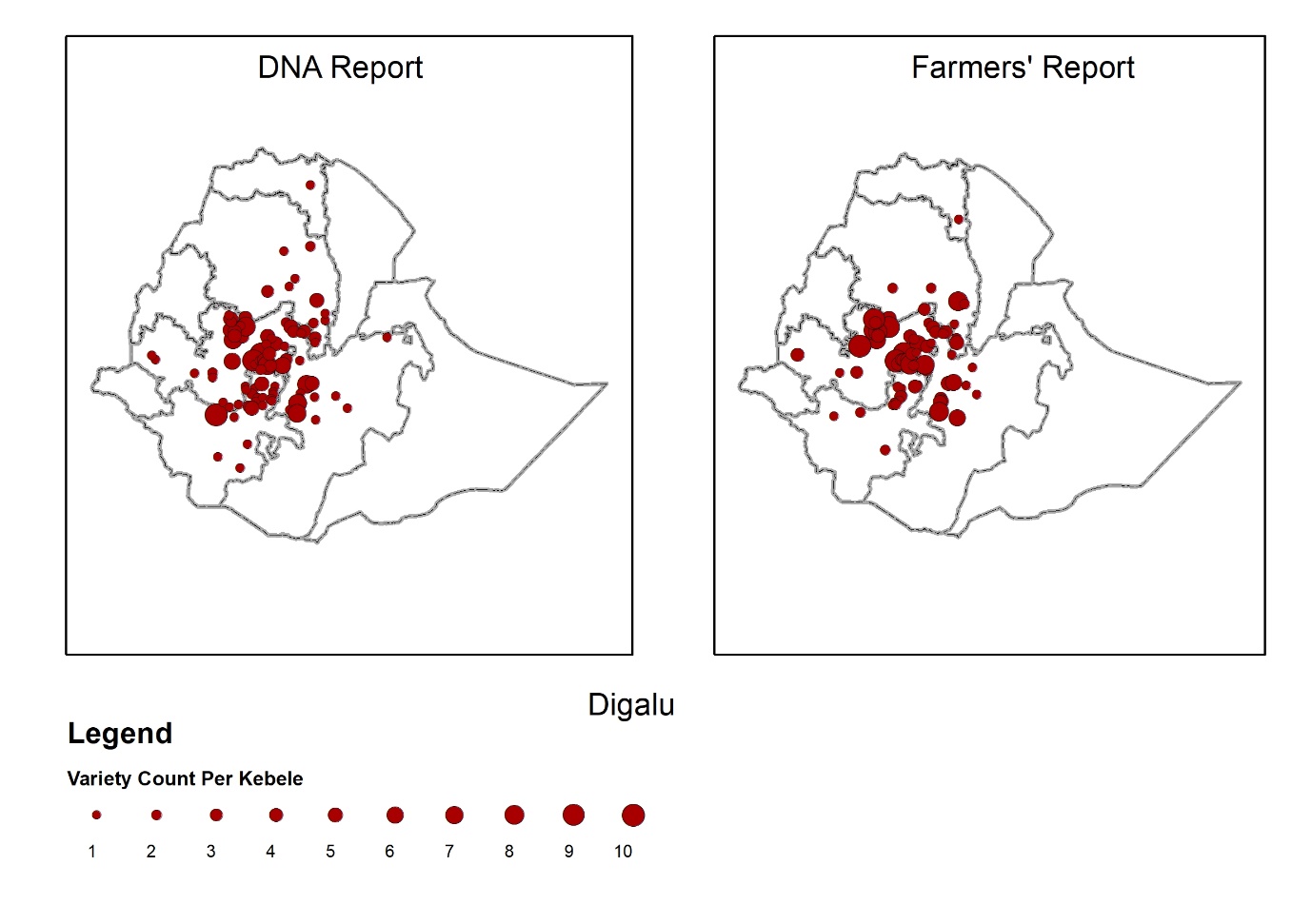
**

**SI Fig. 3** Geographical distribution of variety ‘*Digalu’* using DNA fingerprinting data (left panels) and farmer reports (right panels; 2016/17, 432 kebeles. Size of dots represent the number of plots per Kebele). [maps generated by YA using ESRI ArcGIS 10.6 https://www.esri.com/en-us/arcgis/products/arcgis-pro/overview]

**
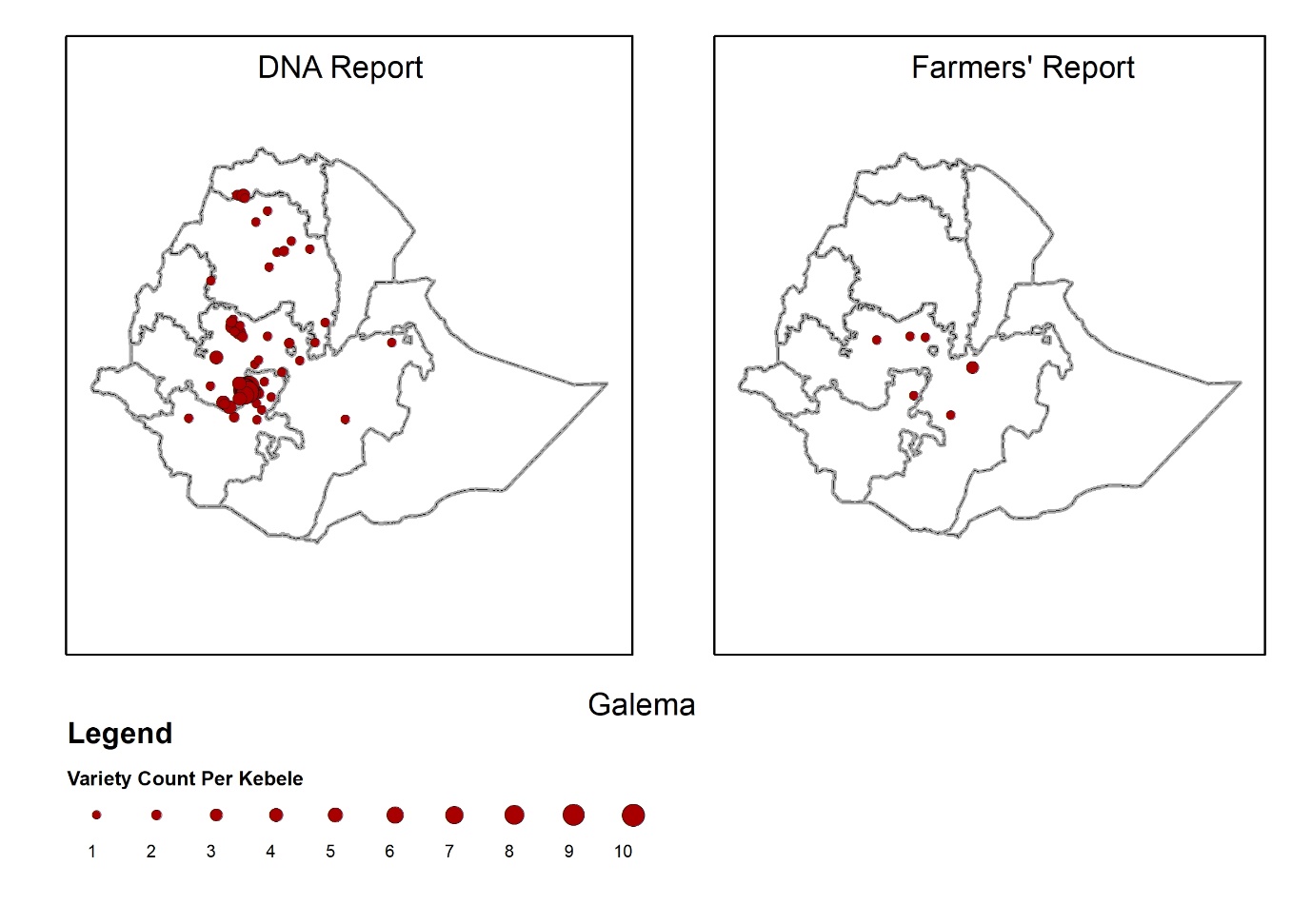
**

**SI Fig 4** Geographical distribution of variety ‘*Galema’* using DNA fingerprinting data (left panels) and farmer reports (right panels; 2016/17, 432 kebeles. Size of dots represent the number of plots per Kebele). [maps generated by YA using ESRI ArcGIS 10.6 https://www.esri.com/en-us/arcgis/products/arcgis-pro/overview]

**
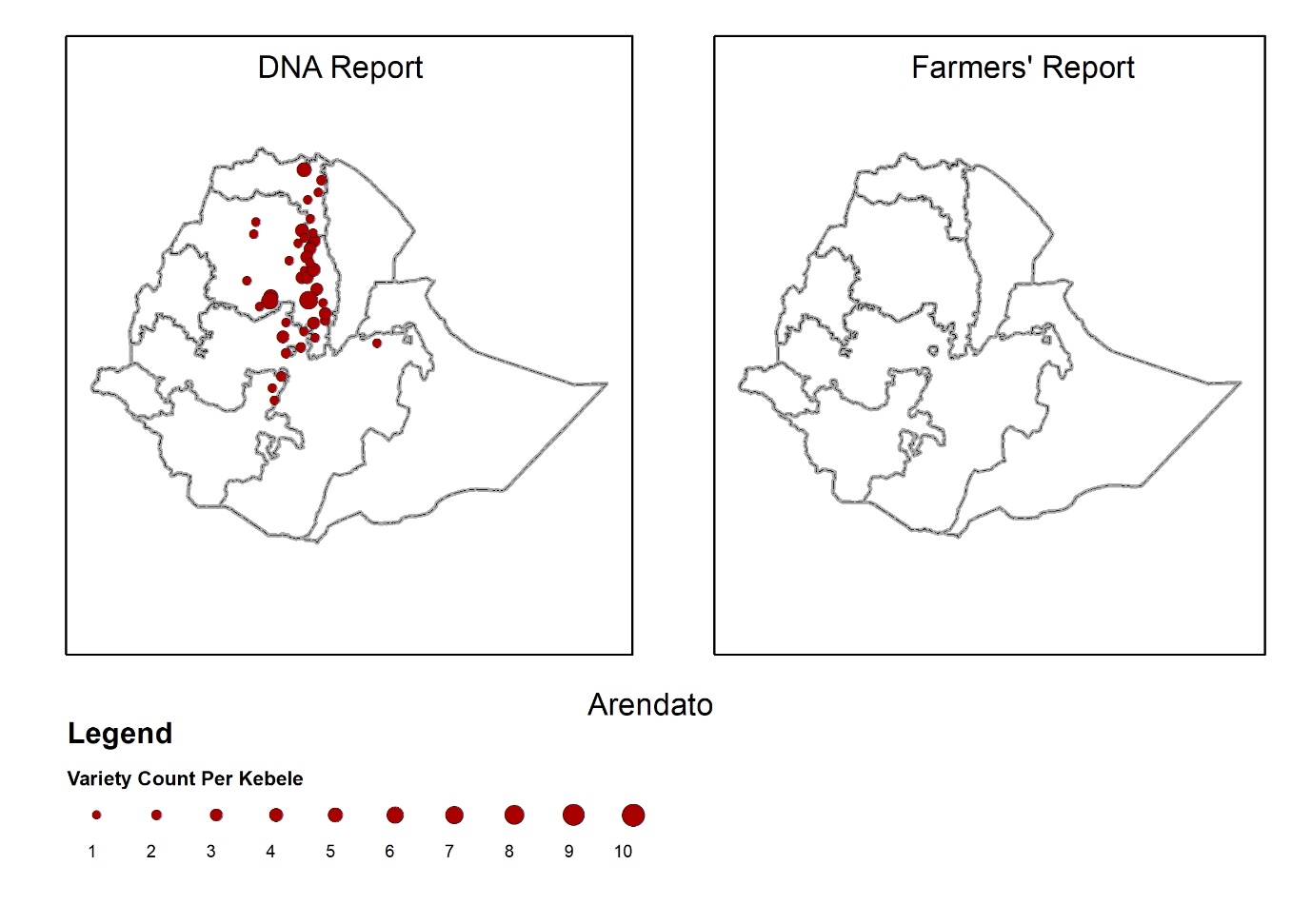
**

**SI Fig 5** Geographical distribution of variety ‘*Arendato’* using DNA fingerprinting data (left panels) and farmer reports (right panels; 2016/17, 432 kebeles. Size of dots represent the number of plots per Kebele). [maps generated by YA using ESRI ArcGIS 10.6 https://www.esri.com/en-us/arcgis/products/arcgis-pro/overview]

**
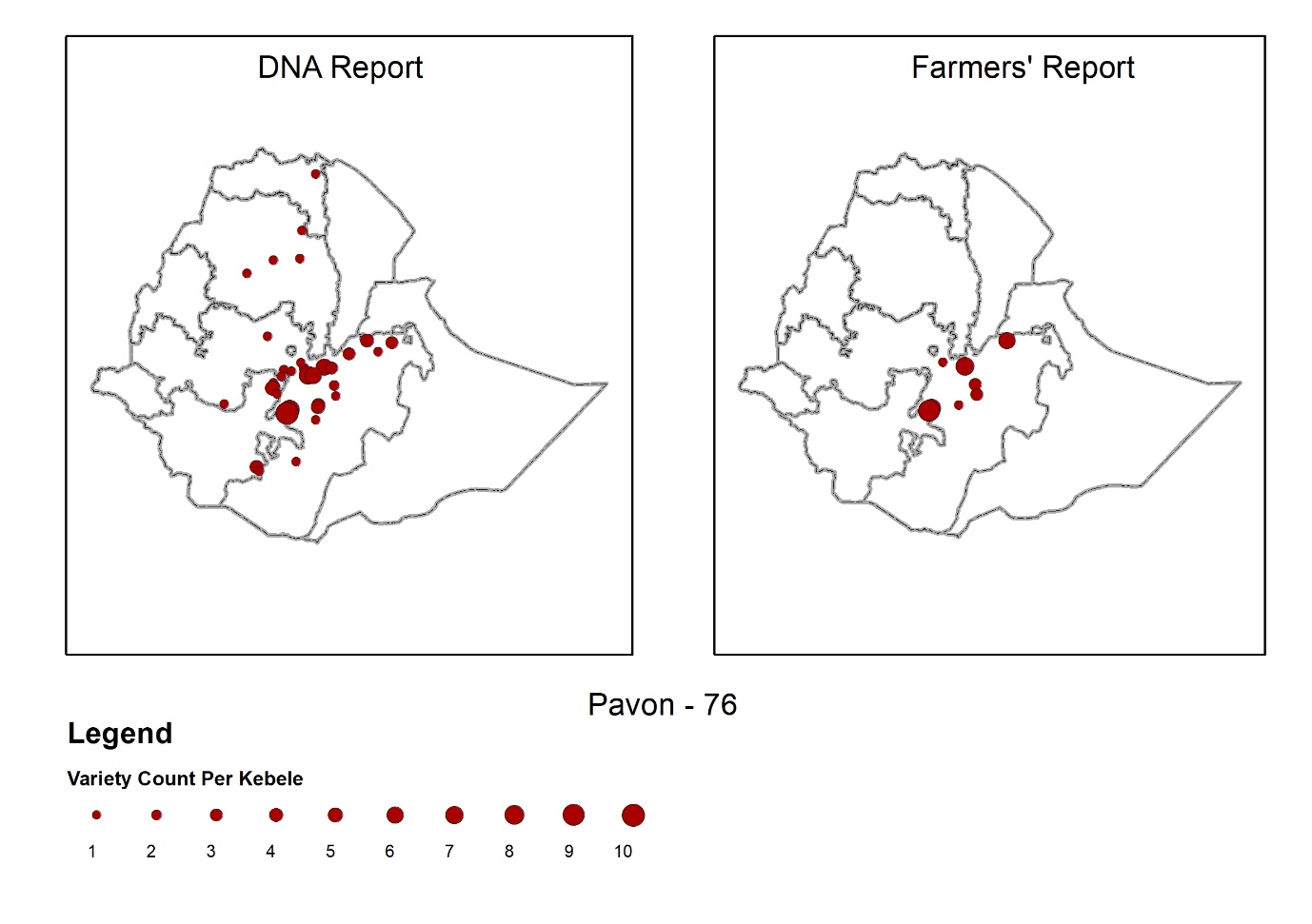
**

**Si Fig 6** Geographical distribution of variety ‘*Pavon-76’* using DNA fingerprinting data (left panels) and farmer reports (right panels; 2016/17, 432 kebeles. Size of dots represent the number of plots per Kebele). [maps generated by YA using ESRI ArcGIS 10.6 https://www.esri.com/en-us/arcgis/products/arcgis-pro/overview]

SI Table 1: Durum wheat varieties identified by DNA fingerprinting and/or farmers’ reports (2016/17 432 kebeles)

| Durum Variety | Year Released | Number of DNA samples Identified | Number of farmer reports |
| --- | --- | --- | --- |
| Arendato | 1967 | 94 | 0 |
| Arsi Robe | 1996 | 0 | 16 |
| Denbi | 2009 | 0 | 1 |
| Ejersa | 2005 | 1 | 2 |
| Laste | 2002 | 6 | 0 |
| Mangudo | 2012 | 2 | 0 |
| Tate | 2009 | 2 | 2 |
| Yerer | 2002 | 2 | 0 |
| Werer-2 | 2009 | 1 | 0 |
| Ude | 2002 | 0 | 1 |
